# Synergistic antitumor effects of combination PI3K/mTOR and MEK inhibition (SAR245409 and pimasertib) in mucinous ovarian carcinoma cells by fluorescence resonance energy transfer imaging

**DOI:** 10.18632/oncotarget.8807

**Published:** 2016-04-18

**Authors:** Kanako Inaba, Katsutoshi Oda, Kazuhiro Aoki, Kenbun Sone, Yuji Ikeda, Aki Miyasaka, Tomoko Kashiyama, Tomohiko Fukuda, Chinami Makii, Takahide Arimoto, Osamu Wada-Hiraike, Kei Kawana, Tetsu Yano, Yutaka Osuga, Tomoyuki Fujii

**Affiliations:** ^1^ Department of Obstetrics and Gynecology, Faculty of Medicine, The University of Tokyo, Tokyo, Japan; ^2^ Imaging Platform for Spatio-Temporal Information, Graduate School of Medicine, Kyoto University, Kyoto, Japan; ^3^ Department of Obstetrics and Gynecology, National Center for Global Health and Medicine, Tokyo, Japan

**Keywords:** ovarian mucinous carcinoma, MAPK pathway, PI3K/mTOR pathway, molecular target therapy, FRET imaging

## Abstract

The aim of this study was to clarify the synergistic effects of dual inhibition of the PI3K/mTOR and MAPK pathways in ovarian mucinous carcinoma (OMC) cells, using fluorescence resonance energy transfer (FRET) imaging. We exposed 6 OMC cell lines to a PI3K/mTOR inhibitor (voxtalisib, SAR245409) and/or a MEK inhibitor (pimasertib), and evaluated synergistic effects using the Chou–Talalay method. Then, S6K (PI3K pathway) and ERK (MAPK pathway) kinase activities, and their individual proliferative or cytotoxic effects were calculated by time-lapse FRET imaging. In combination with SAR245409, pimasertib (30 nM) synergistically inhibited cell growth (combination indexes: 0.03–0.5) and induced apoptosis in all 6 OMC cell lines. FRET-imaging results demonstrated that ERK inhibition induced both anti-proliferation and apoptosis in a dose-dependent manner in both MCAS and OAW42 cells. However, S6K inhibition suppressed proliferation in a threshold manner in both cell lines, although apoptosis was only induced in OAW42 cells. These results demonstrated that combined PI3K/mTOR and MEK inhibition exhibited synergistic antitumor effects in OMC cells and that FRET imaging is useful for analyzing kinase activities in live cells and elucidating their cytostatic and cytotoxic effects.

## INTRODUCTION

Ovarian cancer is a leading cause of death among patients with gynecologic malignancies [1, 2]. Ovarian mucinous carcinomas (OMC) accounts for ~10% of epithelial ovarian carcinomas [3]. Although a high proportion of OMCs are confined to the ovary and cured mainly by surgical excision, patients with advanced OMC have a poorer prognosis due to low sensitivity to platinum-based chemotherapy [4–7]. The mechanism of chemoresistance remains unknown, and chemotherapy-based clinical trials are difficult to perform due to limited participants. Therefore, novel treatment options for advanced or recurrent OMC should be established based on the tumor biology. However, limited studies have related to targeted molecular therapies against OMC [7, 8]. The mitogen-activated protein kinase (MAPK) pathway might represent one possible target in OMC, because mutations in the *KRAS* GTPase gene are frequent in OMC (50–60%) [9], and exome-level sequencing studies in OMC revealed various genetic alterations in the MAPK pathway [10]. Although phosphatidylinositol 3-kinase (PI3K)-activating mutations, such as *PIK3CA* and *PTEN*, are rare (<10%) in OMC [11], *KRAS* mutations can also activate the PI3K/mammalian target of rapamycin (mTOR) pathway [12]. Accordingly, a PI3K/mTOR inhibitor, NVP-BEZ235, suppressed cell proliferation in OMC cell lines [8]. In addition, co-targeting the PI3K/mTOR and MAPK pathways synergistically inhibited the growth of various ovarian cancer cell lines [13]. However, the antitumor effects of these drugs vary significantly among cancer types [14], which might relate to the complexity of the signaling networks [15, 16]. We recently reported that combination treatment with a PI3K/mTOR inhibitor, SAR245409 (voxtalisib), and a MEK inhibitor, pimasertib, showed synergistic antitumor effects in 6 out of 12 endometrial cancer cell lines and that mutational statuses of *KRAS*, *PIK3CA*, and *PTEN* were not involved [17]. Pimasertib, alone or in combination with SAR245409, is currently being investigated in Phase I–II trials. Collectively, these findings suggest that co-targeting the PI3K/mTOR and MAPK pathways might be a therapeutic option for certain OMC cells and that the synergy of dual inhibition might differ among cell lines, even within the same OMC histological types.

Quantitative monitoring of intracellular signaling in living cells is enabled by recent advances in biosensors, based on fluorescence resonance energy transfer (FRET). To date, FRET biosensors have enabled visualization of a wide range of cellular events such as protein kinase activities, protein-protein interactions, and second-messenger activities [18, 19]. Using FRET biosensors for ERK and S6K, we demonstrated differences in sensitivity to MEK and PI3K inhibitors in *KRAS-* and *BRAF-*mutant lung or colorectal cancer cell lines [20, 21]. However, FRET imaging has not yet been applied to ovarian cancers, including OMC.

Here, we evaluated the synergistic antitumor effects of combination treatment with a PI3K/mTOR inhibitor (SAR245409) and a MEK inhibitor, pimasertib, in OMC cells. We also quantified the activity of S6K (in the PI3K/mTOR pathway) and ERK (in the MAPK pathway) in OMC cells under various treatment concentrations and durations via FRET imaging. Finally, we derived a mathematical model to directly associate the kinase activities of S6K or ERK with the anti-tumor effects (cytostatic and/or cytotoxic) of these compounds in OMC cells.

## RESULTS

### Antiproliferative effect of SAR245409 and pimasertib in OMC cell lines

**Figure 1:**
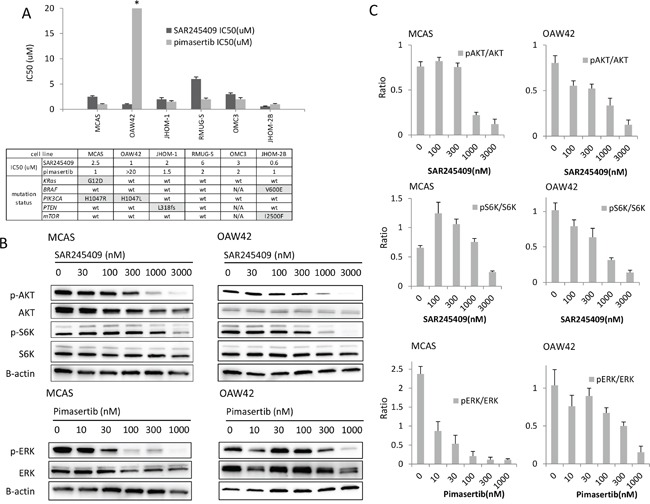
Inhibition of cell proliferation by SAR245409 and pimasertib **A.** Calculation of the IC_50_ values of SAR245409 and pimasertib according to MTT assay data. The results are shown as the mean ± SE of 3 independent experiments. The IC_50_ of pimasertib for OAW42 cells was >20 μM. The table shows the mutation statuses of each cell line. **B.** Western blot analysis of MCAS and OAW42 cell lysates, following treatment with SAR245409 (0–3,000 nM) or pimasertib (0–1,000 nM) for 3 h. p-AKT, p-S6K, and p-ERK levels were evaluated to assess suppression of the PI3K, mTOR, and MAPK pathways, respectively. **C.** Quantified ratios of p-AKT and p-S6 to total AKT and S6 protein levels in response to SAR245409, as well as p-ERK levels in response to pimasertib. Levels were quantified using Image J software. The results are shown as the mean ± SE of 3 independent experiments.

We evaluated the anti-tumor effects of each single agent, SAR245409 and pimasertib, in 6 OMC cell lines by performing methyl thiazolyl tetrazolium (MTT) assays. The mutational statuses of *PIK3CA*, *PTEN*, and *mTOR* (PI3K-pathway genes) and *KRAS* and *BRAF* (MAPK-pathway genes) are shown in Figure 1A. MCAS cells harbor mutations in both *KRAS* and *PIK3CA*, and JHOM-2B cells harbor mutations in both *BRAF* and *mTOR*. OAW42 and JHOM-1 cells harbor a *PIK3CA* and *PTEN* mutation, respectively. The half-maximal inhibitory concentration (IC_50_) values of SAR245409 and pimasertib varied from 0.6 to 6 μM and 1.0 to >20 μM, respectively (Figure 1A). Although the IC_50_ of pimasertib in OAW42 was higher than those in the other 5 cell lines, no significant difference in pimasertib sensitivity was observed among the other 5 lines.

The effects of SAR245409 and pimasertib on each target pathway were evaluated by immunoblotting (Figure 1B), and the phosphorylation levels of the target proteins were quantified using Image J software (Figure 1C). In MCAS and OAW42 OMC cells, 1 μM SAR245409 or higher was required to suppress the phosphorylation of AKT (Ser473, p-AKT) and S6K (Thr389, p-S6K), and a 30–300 nM or higher dose of pimasertib suppressed ERK phosphorylation (p-ERK). Overall, the IC_50_ values of the PI3K/mTOR- and MEK-pathway inhibitors were much higher than the minimum doses required to suppress phosphorylation of their target proteins, suggesting that inhibition of either pathway alone might be insufficient to inhibit cell proliferation.

### Synergistic effects of the combination of SAR245409 and pimasertib

**Figure 2:**
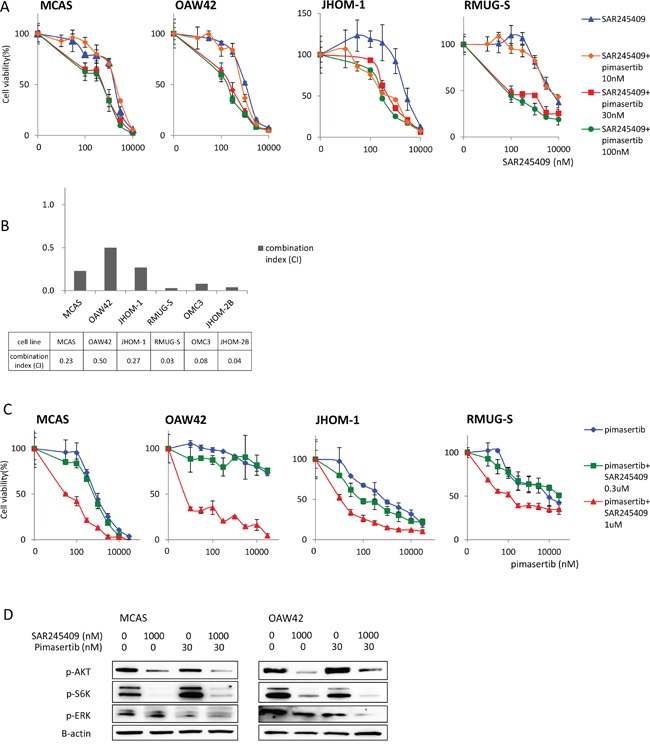
Enhanced antitumor effects of combination treatment with SAR245409 and pimasertib **A.** MTT assays following combination SAR245409 and pimasertib treatment. The pimasertib concentration was fixed at 100, 30, or 10 nM, and the data were compared with those following treatment with SAR245409 alone. The results are shown as the mean ± SE of 3 independent experiments. **B.** CIs were calculated using the Chou–Talalay method. Each cell line was treated with 30-nM pimasertib and SAR245409 (30–10,000 nM). A CI <1.0 indicated a synergistic effect. **C.** MTT assays following combination therapy. The SAR245409 concentration was fixed at 300 or 1 μM, and the data were compared with those obtained after treatment with pimasertib alone. The results are shown as the mean ± SE of 3 independent experiments. **D.** Phosphorylation of AKT, S6K, and ERK were evaluated by western blotting after treatment with 1-μM SAR245409, 30-nM pimasertib, or a combination thereof.

Next, we examined whether the antitumor effects of SAR245409 and pimasertib were synergistic in combination. Based on immunoblotting data (Figure 1B and 1C), we performed the MTT assays under fixed pimasertib concentrations of 10, 30, or 100 nM, combined with various SAR245409 concentrations (Figure 2A, Supplementary Table 1). The effect of 30-nM pimasertib was comparable with that at 100 nM in all 6 OMC cell lines. However, 10-nM pimasertib combined with SAR245409 did not show an additive effect in any cell lines except for JHOM-1 (Figure 2A, Supplementary Figure 1A). Therefore, we calculated the combination indexes (CIs) using the Talalay–Chou method by adding 30-nM pimasertib with various SAR245409 concentrations to OMC cells. The resultant CIs ranged from 0.03 to 0.50 in these 6 cell lines (Figure 2B), suggesting that the synergistic effect was broadly induced in OMC cells independently of the *KRAS* and *PIK3CA* mutational statuses.

We also performed MTT assays under fixed SAR245409 concentrations (300 nM or 1 μM), in combination with various pimasertib concentrations. The anti-proliferative effect of SAR245409 at 300 nM increased with a low dose of pimasertib (100 nM), and this effect was not further enhanced when combined with a higher dose of pimasertib (>300 nM; blue vs. green line; Figure 2C, Supplementary Table 2). In agreement with the data shown in Figure 2A, cell proliferation was more robustly inhibited by combination treatment with 1-μM SAR245409 compared with that at 300 nM (green vs. red line; Figure 2C, Supplementary Figure 1B).

We then examined whether the combination of 1 μM SAR245409 and 30 nM pimasertib could effectively inhibit the phosphorylation of target proteins in the PI3K and MAPK pathways. AKT and S6K phosphorylation was suppressed by SAR245409 at 1 μM, but not by pimasertib. In addition, ERK phosphorylation decreased by pimasertib at 30 nM in both MCAS and OAW42 cells (Figure 2D). p-ERK levels in OAW42 cells were more robustly suppressed by the combination of SAR245409 at 1 μM and pimasertib at 30 nM, compared with pimasertib at 30 nM alone (Figure 2D).

### Apoptosis induction by combination treatment with SAR245409 and pimasertib

**Figure 3:**
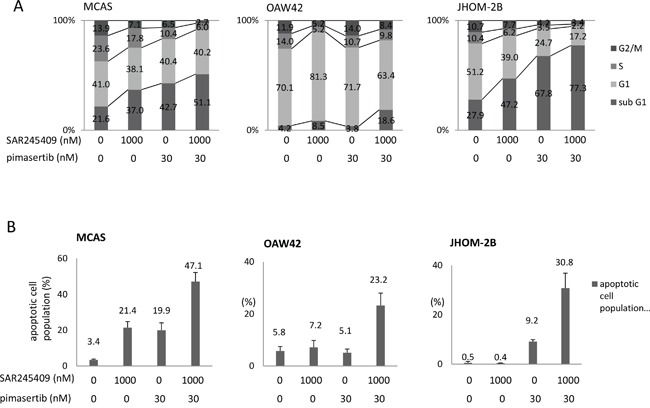
Apoptosis induced by combination SAR245409 and pimasertib treatment **A.** Flow cytometric analysis of the cell cycle in MCAS (left), OAW42 (middle), and JHOM-2B (right) cells treated with 1-μM SAR245409, 30-nM pimasertib, or a combination thereof. **B.** Annexin V-assay results in the 3 cell lines examined in **A.** The results are shown as the mean ± SE of 3 independent experiments.

By flow cytometry, 1-μM SAR245409 with 30-nM pimasertib markedly decreased the S phase population (from 10.4–23.6% to 2.2–6.0%) in all three tested cell lines (MCAS, OAW42, and JHOM-2B), and increased the sub-G1 population in MCAS and JHOM-2B cells (Figure 3A). Furthermore, Annexin V-assay results revealed that this combination markedly increased the ratio of apoptotic cells in all three cell lines (Figure 3B).

### Knockdown of either MEK1/2 or ERK1/2 enhances cell sensitivity to SAR245409

**Figure 4:**
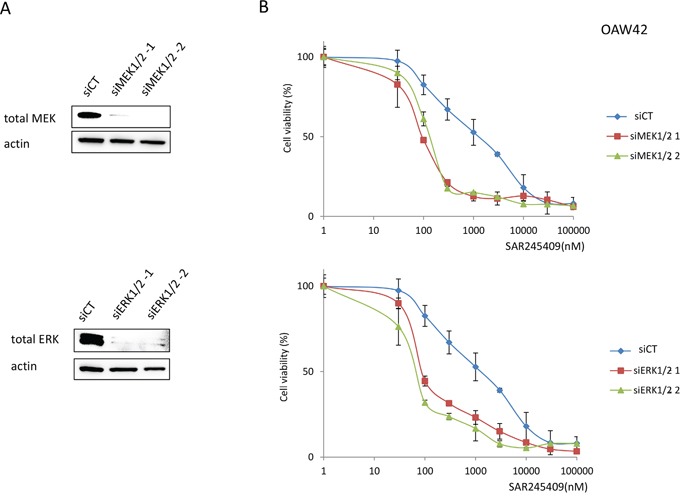
Enhanced sensitivity to SAR245409 by MEK1/2 or ERK1/2 knockdown **A.** Suppression of MEK1/2 or ERK1/2 expression by siRNAs was confirmed by immunoblotting. siRNAs targeting MAP2K1 and MAP2K2, or MAPK3 and MAPK1 (20 nM each) were transfected into OAW42 cells in various combinations. (i) siMEK1/2-1, (ii) siMEK1/2-2, (iii) siERK1/2-1 and (iv) siERK1/2-2 correspond to mixtures of (i) HSS108559 and HSS183388, (ii) HSS108560 and HSS183389, (iii) HSS108538 and HSS183535, and (iv) HSS108539 and HSS183536, respectively. Non-silencing siRNA (siCT) was used as a control. **B.** MTT assays were performed in OAW42 cells under exposure to SAR245409 (30–100,000 nM) following lipofection with siRNAs to either MEK1/2 or ERK1/2. The results are shown as the mean ± SE of 3 independent experiments.

To confirm the effect of inhibiting the MAPK pathway with combination treatment, we silenced MEK 1/2 or ERK1/2 in OAW42 cells before treatment with various SAR245409 concentrations. MEK 1/2 and ERK1/2 expression were suppressed by >80% using two small-interfering RNAs (siRNAs) against each gene (Figure 4A). SAR245409 and either MEK 1/2 or ERK1/2 gene silencing significantly suppressed cell proliferation, compared to SAR245409 treatment alone (relative to SAR245409 and the negative control siRNA) in OAW42 cells (Figure 4B, Supplementary Table 3).

### S6K- and ERK-activity quantification by FRET imaging

**Figure 5:**
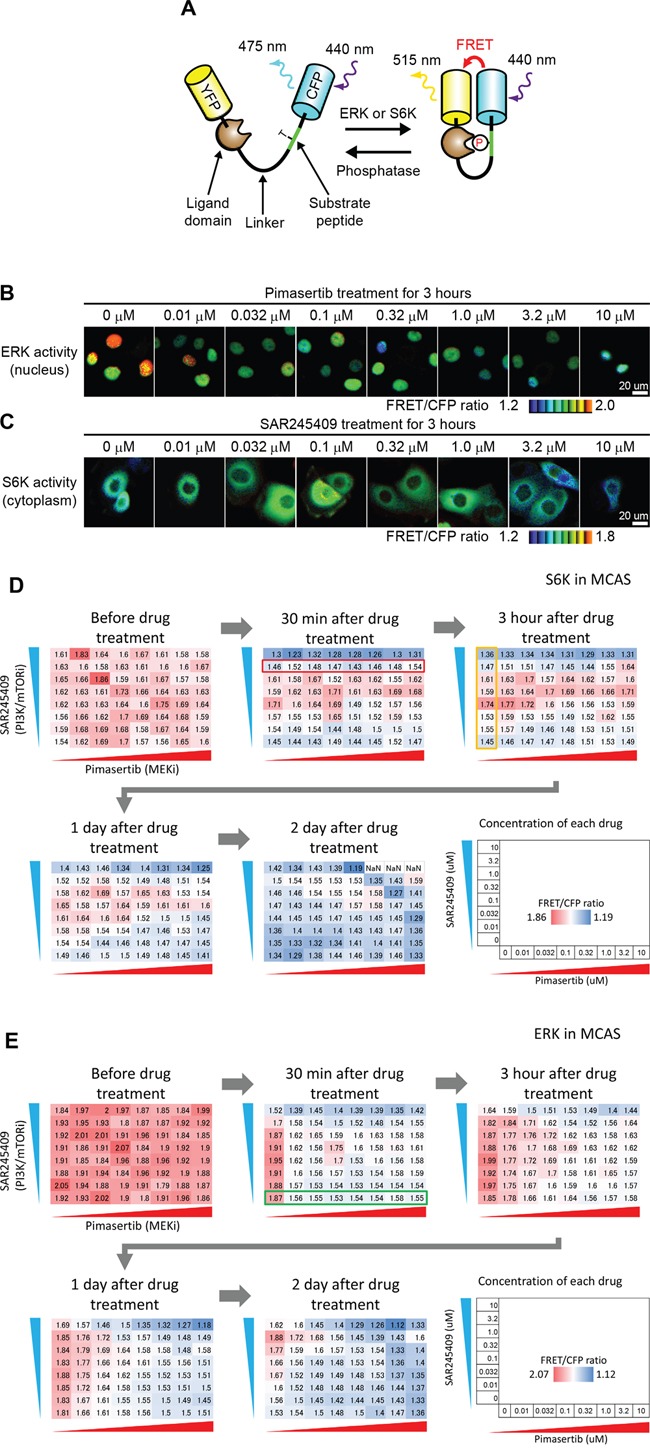
S6K and ERK activity observed by time-lapse FRET imaging **A.** FRET biosensor scheme. A biosensor is phosphorylated by endogenous ERK or S6K, leading to an increase in FRET. **B, C.** MCAS cells stably expressing ERK (B) or S6K (C) FRET biosensors were treated with the indicated concentration of pimasertib (B) or SAR245409 (C) for 3 h. **D.** S6K activity of MCAS cells treated with SAR245409 and pimasertib at various concentrations was quantified before, and at 30 min, 3 h, 1 day, and 2 days post-treatment. The concentrations of each drug are indicated. The values in each panel demonstrate the average of FRET/CFP ratio (N > 10 cells), indicating S6K activity. Red and blue colors represent higher and lower S6K activity, respectively. The results shown are representative of two independent experiments. **E.** The ERK activity of MCAS cells treated with SAR245409 and pimasertib as in panel D. The values in each panel demonstrate the average of the FRET/CFP ratio (N > 10 cells), indicating ERK activity. Red and blue colors represent higher and lower ERK activity, respectively. The results shown are representative of two independent experiments.

We employed FRET biosensors for ERK and S6K, namely EKAREV-NLS and Eevee-S6K-NES, respectively (Figure 5A). These biosensors are phosphorylated by endogenous ERK or S6K, leading to intramolecular binding between sensor and ligand domains. The associated conformational change brings YFP in close proximity to CFP, allowing FRET [22]. We generated cell lines stably expressing either the ERK or S6K FRET biosensor in MCAS and OAW42 cells using a transposon system. MCAS cells expressing EKAREV-NLS (Figure 5B) or Eevee-S6K-NES (Figure 5C) were treated with pimasertib and SAR245409 for 3 h. Pimasertib dose-dependently suppressed ERK activity, which correlated with FRET/CFP ratios (Figure 5B), in agreement with the immunoblotting data (Figure 1B, 1C, and 2D), showing that p-ERK activity was suppressed by 30–100 nM or higher pimasertib doses. S6K activity in MCAS cells exhibited a bell-shaped response to SAR245409; i.e., a low dose of SAR245409 (~100 nM) preferentially activated S6K activity, and a high dose suppressed S6K activity (Figure 5C). These data were consistent with the biochemical data (Figure 1C, middle and lower left), validating the use of these FRET biosensors for quantitative analysis.

Employing FRET imaging in living cells, we quantified ERK and S6K activity in MCAS (Figure 5D, 5E) and OAW42 (Supplementary Figure 2A, 2B) cells, which were seeded on 96-well glass-bottom plates and treated with 64 different combinations of pimasertib and SAR245409 concentrations for 0 min, 30 min, 3 h, 1 day, or 2 days. S6K was activated at a low dose of SAR245409 (0.1 nM) from 30 min to 1 day in MCAS cells (Figure 5D). The observed S6K inhibition at a high pimasertib dose (> 0.32 μM) on day 1 (Figure 5D) agreed with our previous finding of mTOR upregulation via ERK-dependent transcription in KRAS-mutant cancer cells [21]. Here, ERK activity was clearly inhibited 30 min after treatment with a low pimasertib dose (0.032–0.32 μM), but was reactivated at 3 h and later after treatment (Figure 5E). This response might be due to negative feedback from ERK to Sos, Raf, and/or MEK [23–26]. A high SAR245409 dose (10 μM) also inhibited ERK activity at each time point (Figure 5E). This suppression was potentially caused by crosstalk between the MAPK and PI3K/mTOR pathways; either pathway's activity can be suppressed especially when using high-concentration chemical inhibitors [27]. Both S6K and ERK activities in cultured cells were slightly reduced at 48 h even in the absence of SAR245409 and pimasertib (Figure 5D and 5E, 2 days), which might have been caused by contact inhibition.

### Roles of S6K and ERK activity in cell proliferation and cell death

**Figure 6:**
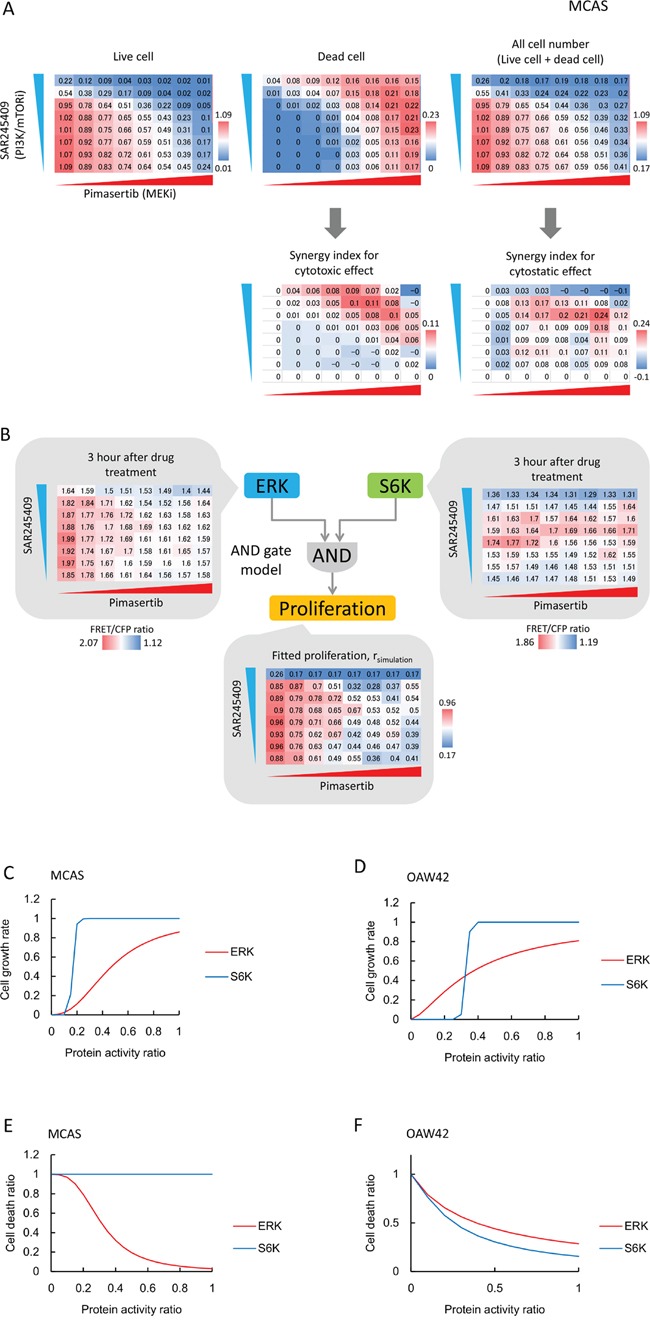
S6K and ERK roles in cell proliferation and cell death are cell-line dependent **A.** Cytostatic and cytotoxic effects of SAR245409 and/or pimasertib treatment in MCAS cells were measured by MTT (upper left) and LDH (upper middle) assays. SAR245409 and/or pimasertib were used as described in Figure 5. Cell numbers were calculated based on the sum of live and dead cells (upper right). Values were normalized to the number of control cells following DMSO treatment. Red and blue colors represent higher and lower values, respectively. The results represent the averaged values of 3 independent experiments. The synergy index was calculated using Bliss independence. **B.** Cell-proliferation data (Figure 6A) in MCAS cells were fitted with ERK- and S6K-activity data, based on the “AND gate” model. **C, D.** Contributions of ERK (red lines) and S6K (blue lines) activity to cell proliferation in MCAC (C) and OAW42 (D) cells are plotted based on mathematical models and parameter estimations. **E, F.** Contributions of ERK (red lines) and S6K (blue lines) activity to cell death in MCAC (E) and OAW42 (F) cells are plotted, based on mathematical models and parameter estimations.

To link ERK and S6K activity to cancer cell phenotypes (proliferation and cell death), we measured the numbers of live and dead cells in MTT and lactate dehydrogenase (LDH) assays 2 days after inhibitor treatments under the conditions described for Figure 5D and 5E. The numbers of live and dead cells were normalized to values from reference wells. SAR245409 or pimasertib treatment alone showed cytostatic and cytotoxic effects, and the combination showed synergistic effects in MCAS cells (Figure 6A). In OAW42 cells, SAR245409 treatment induced more potent cytostatic and cytotoxic effects than pimasertib (Supplementary Figure 3).

Finally, we combined the cell-proliferation and cell-death data (Figure 6A and Supplementary Figure 3) from the FRET-imaging, using a mathematical model to evaluate the contributions of S6K and ERK to cell proliferation and cell death in MCAS and OAW42 cells. We derived an “AND gate” model, in which the multiplication of ERK and S6K activities dictated the proliferation and cell death (Figure 6B). After parameter fitting, we obtained Hill functions for the ERK and S6K activities toward proliferation and cell death. ERK and S6K activity linearly augmented MCAS and OAW42 cell proliferation (Figure 6C, 6D). Comparatively, S6K activity promoted cell proliferation in a threshold manner. Notably, the threshold value of S6K in OAW42 cells (~0.3) was higher than that in MCAS cells (~0.15), indicating that OAW42 cells were more sensitive to the PI3K/mTOR inhibitor (Figure 6C, 6D). Figure 6E and 6F show the cell-death levels at the indicated S6K and ERK activities. In MCAS cells, lower ERK activity induced cell death with relatively high cooperativity, whereas S6K activity did not promote cell death (Figure 6E). Therefore, ERK activity should be inhibited to induce cytotoxicity in MCAS cells. However, inhibition of both ERK and S6K activities was required to induce cell death in OAW42 cells (Figure 6F). Collectively, these results demonstrated quantitative differences of sensitivities to MEK or PI3K/mTOR inhibitors between MCAS and OAW42 cells.

## DISCUSSION

Here, we found that (i) combination PI3K/mTOR- and MEK inhibitor-treatment synergistically suppressed cell growth in OMC cell lines; (ii) the synergistic effect was dependent on both cytostatic and cytotoxic effects; and (iii) multi-well FRET imaging and mathematical modeling revealed that S6K and ERK activities contributed to the cytostatic or cytotoxic effects.

Combination therapy with SAR245409 and pimasertib induced synergistic antitumor effects in all 6 OMC cell lines tested. Considering the low chemosensitivity of OMC, combination therapy might be promising for advanced or recurrent OMC patients. The IC_50_ values for pimasertib alone were much higher (>1 μM) than the doses required to suppress p-ERK production (30–300 nM). Therefore, the PI3K/mTOR pathway was considered essential for cell proliferation and/or cell survival. Synergistic effects mediated by inhibiting both the PI3K and the MAPK pathways have been reported for several types of cancers [28–30], and activation of the RAS/MAPK pathway may be associated with the synergistic effects of the PI3K/mTOR- and MEK-pathway inhibitors [31, 32]. Our data support the importance of inhibiting the PI3K/mTOR and MAPK pathways in OMC cells. Although the mutation rates of the PI3K-pathway genes were generally low in OMC clinical samples [11], the PI3K/mTOR pathway mutations (*PIK3CA*, *PTEN*, or *mTOR*) were identified in 3 of 6 OMC cells. Here, the IC_50_ values were ≤2.5 μM for pimasertib in cells with *KRAS* or *BRAF* mutations, and were ≤2.0 μM for SAR245309 in cells with *PIK3CA*, *PTEN*, or *mTOR* mutations. Therefore, the sensitivity of these compounds may reflect the mutational status. As ERK1/2 knockdown increased SAR245409 sensitivity comparably to that observed following MEK1/2 knockdown, ERK inhibition would be essential for synergistically co-targeting the PI3K and MAPK pathways. It should be further clarified how the activity of each component of the PI3K/mTOR and MAPK pathways promotes sensitivity to single or combination treatment with PI3K/mTOR and MEK inhibitors.

Combination treatment markedly induced apoptosis in OMC cells. As OMC cells are generally resistant to conventional chemotherapy, synergistic cytotoxicity of combination treatment (as with SAR245409 and pimasertib) might be a promising approach for this cancer. The combination treatment also increased the population of G2/M phase cells. Therefore, this combination induced both cytotoxic and cytostatic effects. As both the MAPK and PI3K pathways are broadly activated in ovarian endometrioid, clear cell, and low-grade serous carcinomas, this combination might have synergistic antitumor effects on other histological types of ovarian cancer [9, 33].

Using FRET-imaging and mathematical modeling, we quantitated the differing cytostatic and cytotoxic effects following combination therapy in OMC cell lines. Time-lapse FRET imaging was previously employed in several cancer types [34–36]. However, this is the first report of the application of FRET biosensors to analyze molecularly targeted drugs in ovarian cancer cell lines. FRET-imaging systems and mathematical modeling enabled us to investigate (i) how treatment with molecularly targeted drugs alone or in combination affected target kinase activities with various durations and dosages in live cells in a single assay, (ii) how the targeted kinases affected each other through feedback and/or feedforward regulation, and (iii) how each kinase activity contributed to cell proliferation and cell death. In two cell lines, the ERK inhibition blocked cellular proliferation and induced cell death, both of which were almost linearly associated with ERK activity. In contrast, S6K activity inhibited cell proliferation in a threshold manner. ERK inhibition slowly reduced S6K activity, which may reflect the synergistic effects of combination treatment with MEK and PI3K/mTOR inhibitors. Although the inhibition of S6K activity was indispensable for inducing cell death in OAW42 cells, it did not show any dose-dependent effect on MCAS cell proliferation. These results are consistent with the mutational status of the *KRAS* or *PIK3CA* genes; namely, MCAS cells harbor *KRAS* and *PIK3CA* mutations and exhibit greater sensitivity to MEK inhibitors than to PI3K/mTOR inhibitors. In contrast, OAW42 cells harbor only *PIK3CA* mutations, suggesting a higher sensitivity to PI3K/mTOR inhibitors than to MEK inhibitors. Future studies and careful consideration will be needed to evaluate the relationship between mutation statuses and the effects of molecularly targeted drugs, as FRET reflects both exogenous and endogenous kinase activities.

Our study has several limitations. First, the synergistic mechanisms between SAR245409 and pimasertib, as well as the cytotoxic mechanisms elicited by inhibiting the associated pathways, were not fully elucidated. Second, time-lapse FRET imaging was analyzed in only two cell lines, and further study is warranted to clarify similarities and differences among various OMC cell lines. Third, *in vivo* preclinical studies and/or clinical trials are required to evaluate the synergistic effects and feasibility of this combination therapy in OMC. However, good efficacy and feasibility of SAR245409 and pimasertib have been reported in clinical trials and mouse xenograft models [37, 38]. The maximum-tolerated doses for the combination are within the range of the active doses of each drug as monotherapies in clinical trials [37], and the combination showed robust antitumor activity without significant additive effects in an ovarian cancer-xenograft mouse model [38].

In summary, our results demonstrated that combination therapy with SAR245409 and pimasertib synergistically induced cytotoxic and cytostatic responses in OMC cells. We also found that activation of both the PI3K and MAPK pathways is essential for these effects, and that their relative contributions might vary among cell types. We also showed that FRET-imaging systems can be useful for analyzing the mechanisms of action of each drug in combination therapies against cancer cells.

## MATERIALS AND METHODS

### Cell lines and inhibitors

MCAS and OAW42 cells were purchased directly from the American Type Culture Collection (Manassas, VA, USA). JHOM-1, JHOM-2B, OMC3, and RMUG-S cells were purchased directly from the Riken BioResource Center (Ibaragi, Japan). Both cell banks routinely characterize their cell lines. Assays completed with these cell lines were completed in our laboratory within 6 months after resuscitation. MCAS and OAW42 were maintained in Dulbecco's Modified Eagle Medium (DMEM) with 10% fetal bovine serum (FBS), and JHOM-2B cells were maintained in DMEM: Nutrient mixture F12 (DMEM/F12) with 10% FBS. The remaining cell lines were maintained in F12 with 10% FBS. SAR245409 and pimasertib were provided by Sanofi (Paris, France) and Merck Serono (Darmstadt, Germany), respectively. SAR245409 is a highly selective and potent ATP-competitive inhibitor of pan-class I PI3Ks and mTORC1/mTORC2 [17, 39]. Pimasertib is a highly potent ATP-noncompetitive second-generation inhibitor of MEK1 and MEK2. Pharmacological information regarding SAR245409 and pimasertib including molecular-structure and preliminary toxicity data have been published [39, 40]. Both agents are orally bioavailable small-molecule inhibitors and have been tested in phase I/II clinical trials for malignant tumors (ClinicalTrials.gov Identifier: NCT01936363). The mutational statuses of *KRAS*, *BRAF*, *PIK3CA*, *PTEN*, and *mTOR* were confirmed by the COSMIC (http://cancer.sanger.ac.uk/cell_lines) and Cancer Cell Line Encyclopedia databases (http://www.broadinstitute.org/ccle/home), and polymerase chain reaction amplification and direct sequencing [13, 41, 42].

### Proliferation and cytotoxicity assays

Cell proliferation was evaluated in MTT assays using the Cell Counting Kit-8 (Dojindo, Tokyo, Japan) or Cell Count Reagents SF (Nacalai Tesque, Kyoto, Japan), both of which employ 2-(2-methoxy-4-nitrophenyl)-3-(4-nitrophenyl)-5-(2,4-disulfophenyl) -2H-tetrazolium, monosodium salt [17, 41]. Cells were seeded in 96-well plates at 2 × 10^3^−1 × 10^4^ cells/well and exposed to each drug for 72 h. Cell proliferation was quantified by monitoring changes in the absorbance at 450 nm. Cytotoxicity was measured by LDH release into the culture medium. Briefly, cells were prepared as described for the cell-proliferation assay. The cultured medium (50 μl) was transferred to 96-well plates, and LDH was measured using the CytoTox-ONE Homogeneous Membrane Integrity Assay (Promega, Madison, WI, USA), according to the manufacturer's protocol. The IC_50_ values were determined from the dose-response curves. Each experiment was repeated at least three times.

### Immunoblotting

Cells (5 × 10^5^) in 6-well plates were exposed to SAR245409 and/or pimasertib for the indicated times at the indicated concentrations and lysed as described previously [43]. Primary antibodies against AKT, p-AKT (Ser473), S6K, phosphorylated S6K (p-S6K), ERK, p-ERK (ERK1/2-Thr202/Tyr204) (Cell Signaling Technology, Beverly, MA, USA), and beta-actin (Sigma-Aldrich, St. Louis, MO, USA) were used for immunoblotting [17, 23]. Signals were detected using an immunoblotting system (BioRad Laboratories, Hercules, CA, USA) with ECL Select Detection agents (GE Healthcare, Piscataway, NJ, USA). Immunoblotting results were quantified using Image J software [44].

### Cell cycle analysis

Cells (5 × 10^5^) were seeded in 60-mm dishes and treated with SAR245409 and/or pimasertib for 72 h. Both floating and adherent cells were collected after trypsinization, and cell-cycle distribution was analyzed by flow cytometry (BD FACSCalibur HG, Franklin Lakes, NJ, USA) and CELLQuest pro ver. 3.1 software (Beckman Coulter Epics XL, Brea, CA, USA) [41]. All experiments were repeated three times.

### siRNA transfections

siRNAs were used to inhibit expression of *MAP2K1*, *MAP2K2*, *MAPK3*, and *MAPK1*, encoding MEK1, MEK2, ERK1, and ERK2 proteins, respectively. siRNAs specific to *MAP2K1* (HSS108559, 108560, 108561), *MAP2K2* (HSS183388, 183389, 183390), *MAPK3* (HSS108538, 108539), and *MAPK1* (HSS183535, 183536) were purchased from Invitrogen (Carlsbad, CA, USA). Non-silencing siRNA (siCT) (Stealth RNAi siRNA Negative Control Kit; Invitrogen) was used as a control. OAW42 cells were seeded 24 h before transfection to reach ~30% confluence in 100-mm plates and transfected with 20-nM siRNA duplexes using Lipofectamine RNAiMAX (Invitrogen).

### Statistical analysis

The means ± SEMs from 3 independent experiments were determined. The significance of differences was analyzed by Student's *t*-test, and *p* < 0.05 was considered statistically significant. For combination experiments with SAR245309 and pimasertib, or rapamycin and pimasertib, CIs were calculated according to the Chou–Talalay method, using the IC_50_ values under monotherapy and combination therapy [45]. Synergism, additive effects, and antagonism were defined as CI < 1, CI = 1, and CI > 1, respectively.

### FRET biosensors and the establishment of biosensor-expressing mucinous ovarian cancer cell lines

FRET biosensors for ERK and S6K were developed previously [46]. Stable cell lines expressing ERK and S6K FRET biosensors using a transposon system were established, as described previously [47]. MCAS and OAW42 cells were transfected with pT2Apuro-HistoneH1-mCherry and pCAGGS-T2TP as nuclear markers. After selection with puromycin (Sigma) at 1.0 μg/ml for 7 days, cells were further transfected with pCMV-mPBase and either pPBbsr-EKAREV-nls or pPBbsr-Eevee-S6K-nes, and selected with blasticidin S at a dose of 10 μg/ml for 7 days (InvivoGen, San Diego, CA, USA) to generate cells expressing EKAREV-nls/Histone H1-mCherry or Eevee-S6K/Histone H1-mCherry. Plasmids were transfected using FuGENE HD (Promega) according to the manufacturer's instructions.

### Multi-well FRET imaging

FRET images were obtained and processed as described previously [21, 48]. Briefly, 3,000 cells/well expressing EKAREV-nls/Histone H1-mCherry and Eevee-S6K/Histone H1-mCherry were mixed and plated in 96-well, glass-bottom plates (Asahi Techno Glass; Tokyo, Japan). After attachment, the media was exchanged for 200 μl of imaging medium comprised of Medium 199 (Sigma) with 20 mM HEPES, 10% FBS, and penicillin/streptomycin. Cells were cultured in CO_2_ incubators, and images were captured at the specified time points using an inverted fluorescence microscope (IX83; Olympus, Tokyo, Japan) equipped with a cooled CCD camera (DOC CAM HR; Molecular Devices, Sunnyvale, CA), an illumination system (CoolLED precisExcite; Molecular Devices), an IX2-ZDC2 laser-based autofocusing system (Olympus), a MAC5000 filter wheel controller (Ludl Electronic Products, Hawthorne, NY, USA), an XY stage (SIGMA KOKI, Tokyo, Japan), and an incubation chamber (Tokai Hit, Shizuoka, Japan). The following filters were used for FRET, CFP, and mCherry imaging: 430/24 (Olympus) for FRET and CFP excitation, 572/35 (Olympus) for mCherry excitation, XF2034 dichroic mirror (Omega, Brattleboro, VT, USA) for FRET and CFP, 86006bs dichroic mirror (Chroma, Brattleboro, VT, USA) for mCherry, FF483/32 (Semrock, Rochester, NY, USA) for CFP emission, FF542/27 (Semrock) for FRET emission, and FF01-641/75 (Semrock) for mCherry emission. Cells were imaged with an UPlanSApo ×20 dry objective lens (Olympus). The same four positions were acquired in each well during the time-course experiments. The microscope was controlled by MetaMorph software (Molecular Devices).

### Mathematical modeling

ERK- and S6K-activity data from MCAS and OAW42 cells were utilized as inputs to reproduce cell proliferation, and cell death was utilized as the output. The following equations were derived as an “AND gate” model of ERK and S6K activity:

$${\text{Proliferation}}_{\text{sim}}=\frac{{\text{ERK}}^{{\text{nH}}_{\text{ERK}\_P}}}{{\text{ERK}}^{{\text{nH}}_{\text{ERK}\_P}}+{\text{EC}50}_{\text{ERK}}^{{\text{nH}}_{\text{ERK}\_P}}}\frac{S6K^{{\text{nH}}_{S6K\_P}}}{S6K^{{\text{nH}}_{S6K}}+{\text{EC}50}_{S6K}^{{\text{nH}}_{S6K\_P}}}$$ (eq. 1)

$${\text{Death}}_{\text{sim}}=\frac{{\text{IC}50}_{\text{ERK}}^{{\text{nH}}_{\text{ERK}\_D}}}{{\text{ERK}}^{{\text{nH}}_{\text{ERK}\_D}}+{\text{IC}50}_{\text{ERK}}^{{\text{nH}}_{\text{ERK}\_D}}}\frac{{\text{IC}50}_{S6K}^{{\text{nH}}_{S6K\_D}}}{{S6K}^{{\text{nH}}_{S6K\_D}}+{\text{IC}50}_{S6K}^{{\text{nH}}_{S6K\_D}}}$$ (eq. 2)

where *Proliferation_sim_* and *Death_sim_* indicate the simulated number of total cells and dead cells, respectively (0 < *Proliferation_sim_* < 1, 0 < *Death_sim_* < 1). We also prepared two additional wells having the same cell numbers, without inhibitors, and measured live cells in 1 well in an MTT assay, while we induced cell death with an SDS-containing solution and measured dead cells in LDH assay in the other well. These data demonstrated how many cells were produced in individual wells, indicating effects on cell proliferation, which is inversely related to cytostatic effects. *ERK* and *S6K* represent normalized ERK and S6K activities as measured by FRET imaging 3 h post-inhibitor treatment, respectively (0 < *ERK* < 1, 0 < *S6K* < 1). The fitted parameters are as follows: *nH_ERK_P_* and *nH_S6K_P_* are Hill coefficients for ERK and S6K in *Proliferation_sim_*, respectively. *nH_ERK_D_* and *nH_S6K_D_* are Hill coefficients for ERK and S6K in *Deadh_sim_*, respectively. *EC50_ERK_* and *EC50_S6K_* are the 50% effective concentrations of ERK and S6K for proliferation, respectively. *IC50_ERK_* and *IC50_S6K_* are the 50% inhibitory concentrations of ERK and S6K for cell death, respectively. The parameters were obtained by minimizing the residual sum square (RSS):

$$\text{RSS}=\sum_{i}{\left({\text{Proliferation}}_{\text{ex},i}-{\text{Proliferation}}_{\text{sim},i}\right)}^{2}$$ (eq. 3)

$$\text{RSS}=\sum_{i}{\left({\text{Death}}_{\text{ex},i}-{\text{Death}}_{\text{sim},i}\right)}^{2}$$ (eq. 4)

where *Proliferation_ex_* represents the normalized total cell number, which is the sum of the data of the MTT (live cells) and LDH (dead cells) assays (0 < *Proliferation_ex_* < 1), and *Death_ex_* represents the number of dead cells, measured by the LDH assay. *i* indicates the *i*-th experimental conditions. By optimizing these parameters, the contributions of ERK and S6K to proliferation and cell death were obtained as a Hill function.

## SUPPLEMENTARY FIGURES AND TABLES








