# Supplementary material for: Synergistic antitumor effects of combination PI3K/mTOR and MEK inhibition (SAR245409 and pimasertib) in mucinous ovarian carcinoma cells by fluorescence resonance energy transfer imaging
Source: Oncotarget. 2016 Apr 18;7(20):29577–91. doi: 10.18632/oncotarget.8807 (PMC5045418; doi:10.18632/oncotarget.8807)
Supplement: Supplementary file 1 [file oncotarget-07-29577-s001.pdf]

SUPPLEMENTARY FIGURES AND TABLES

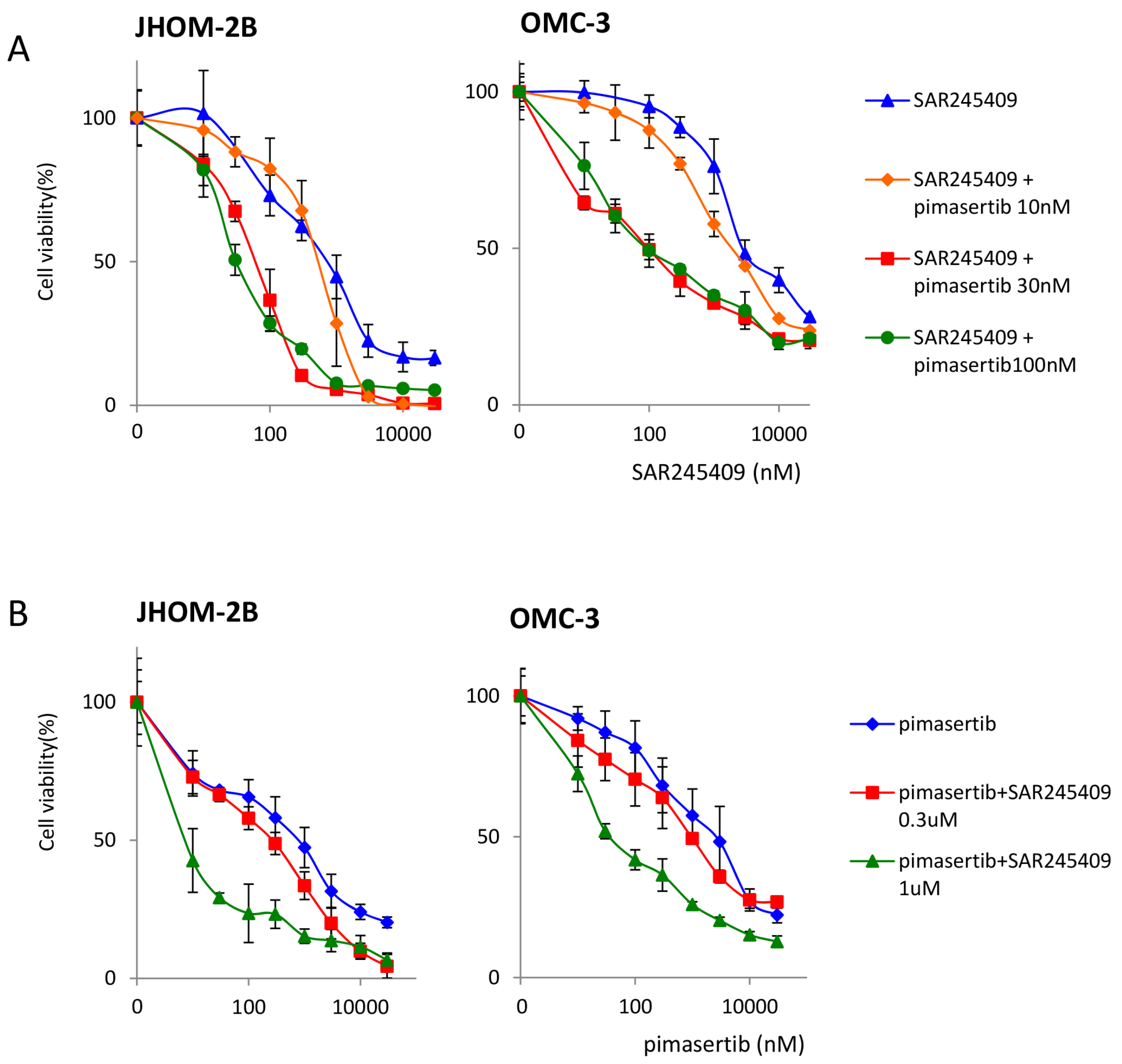

Supplementary Figure S1: MTT assays of the combination of SAR245409 and pimasertib in the remaining 2 cell lines, JHOM-2B and OMC-3. The results are shown as the means ± SE of 3 independent experiments. **A.** The pimasertib concentration was fixed at 100, 30, or 10 nM, and the data were compared with those of SAR245409 alone. **B.** The SAR245409 concentration was fixed at 300 nM or 1 µM, and the data were compared with those of pimasertib alone.

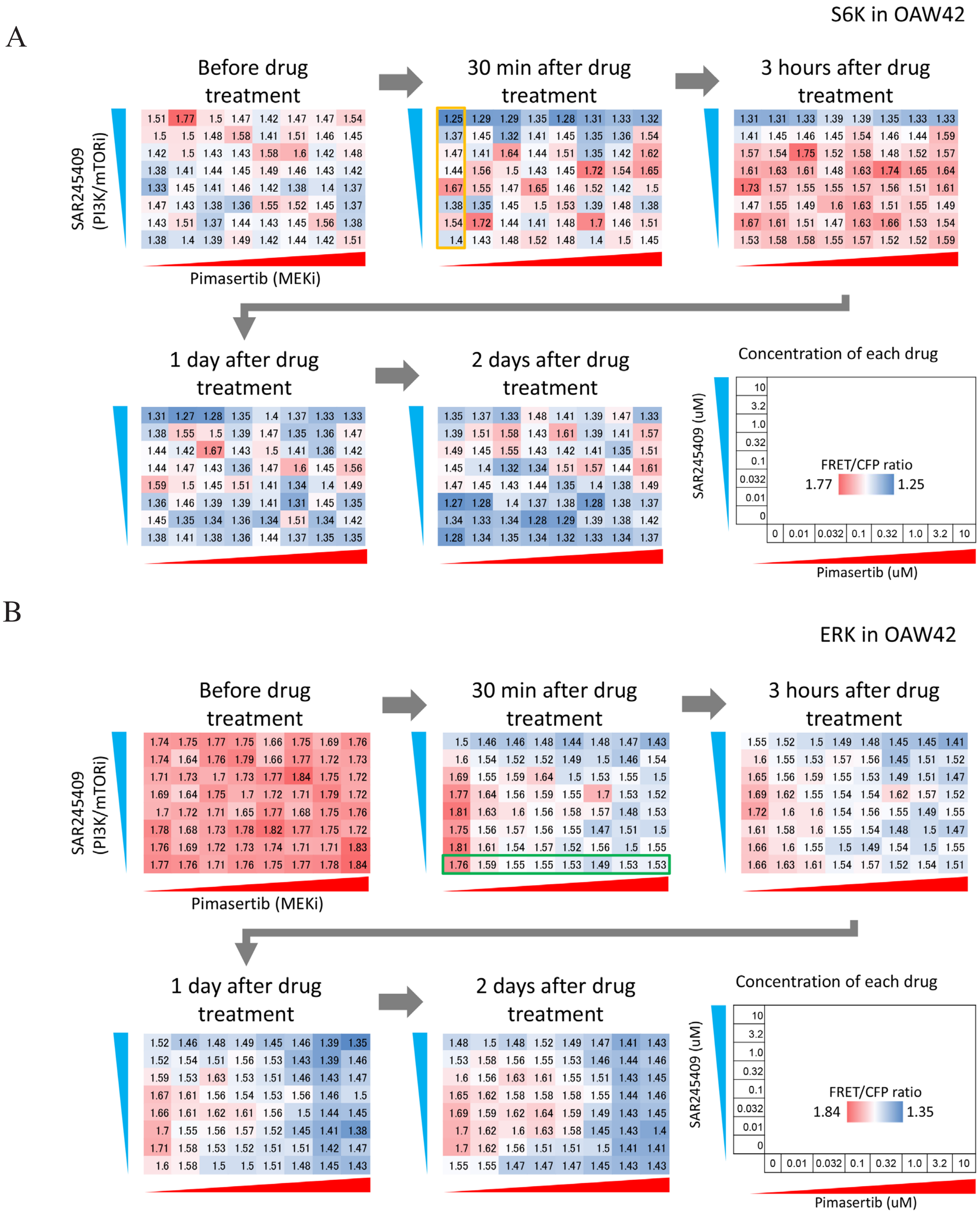

Supplementary Figure S2: S6K and ERK activities observed by time-lapse FRET imaging in OAW42 cells. A. The S6K activity of OAW42 cells treated with SAR245409 and pimasertib at various concentrations was quantified before, 30 min, 3 h, 1 day, and 2 days after treatment. The concentrations of each drug are shown at the lower right. The values in each panel demonstrate the average of the FRET/CFP ratios (N > 10 cells), indicating S6K activity. Red and blue colors represent higher and lower S6K activity, respectively. The results shown are representative data from two independent experiments. **B.** The ERK activity of OAW42 cells treated with SAR245409 and pimasertib as in panel A. The values in each panel demonstrate the average of the FRET/CFP ratios (N > 10 cells), indicating ERK activity. Red and blue colors represent higher and lower S6K activity, respectively. The results shown are representative data from two independent experiments.

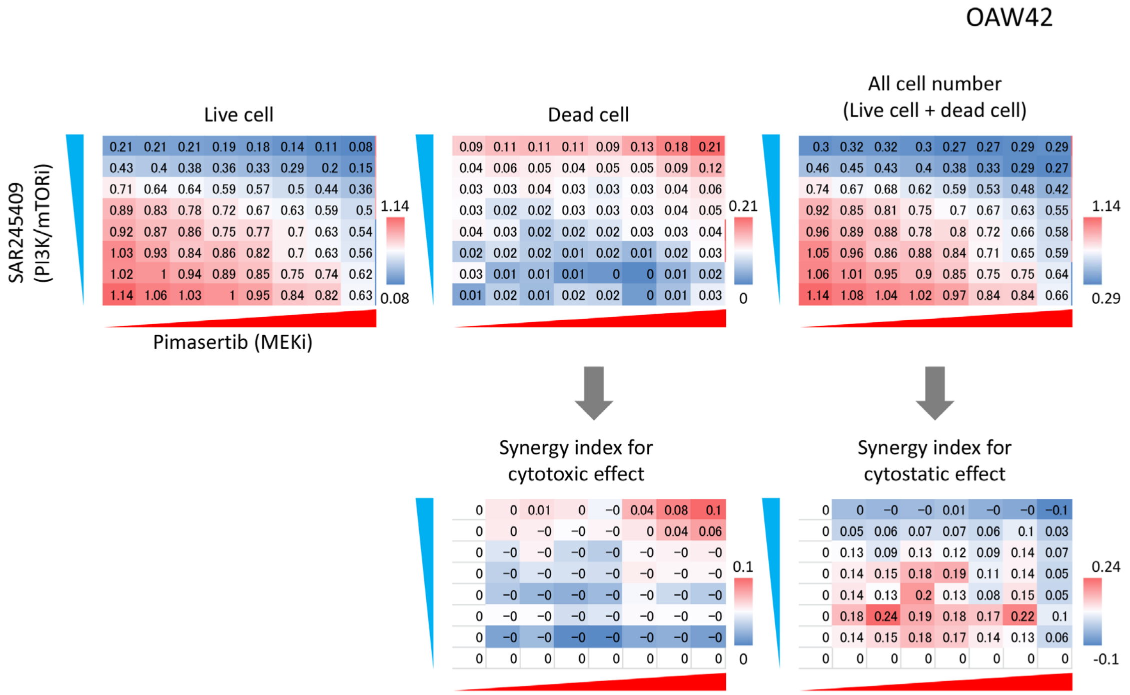

Supplementary Figure S3: Cytotoxic and cytostatic effects of SAR245409 and/or pimasertib treatment in OAW42 cells. Cytostatic and cytotoxic effects of SAR245409 and/or pimasertib treatment in OAW42 cells were measured by MTT (upper left) and LDH (upper middle) assays. SAR245409 and/or pimasertib were applied as in Figure 5. All cell numbers were calculated by the sum of live and dead cells (upper right). The values were normalized by the number of control cells following DMSO treatment. Red and blue colors represent higher and lower values, respectively. The results are the averaged values of 3 independent experiments. The synergy index is calculated using Bliss independence.

Supplementary Table S1: Absorbance of each condition in Figure 2A by MTT assays of combination SAR245409 and pimasertib

See Supplementary File 1

Supplementary Table S2: Absorbance of each condition in Figure 2C by MTT assays of combination SAR245409 and pimasertib

See Supplementary File 2

Supplementary Table S3: Absorbance of each condition in Figure 4B by MTT assays of combination SAR245409 and siRNA to MEK1/2 or ERK1/2

See Supplementary File 3
